# Determinants of the Fiscal Support of Governments in Response to the COVID-19 Pandemic

**DOI:** 10.3389/fpubh.2020.637557

**Published:** 2021-02-05

**Authors:** Sheng-Kun Li, Xueping Liang

**Affiliations:** ^1^School of Business, Henan Normal University, Henan Normal University, Xinxiang, China; ^2^School of Economics, Tianjin University of Commerce, Tianjin University of Commerce, Tianjin, China

**Keywords:** COVID-19 pandemic, COVID-19 uncertainty, world pandemic uncertainty indices, response to the COVID-19 pandemic, fiscal support

## Abstract

Fiscal support measures have different implications for public finances in the near term and beyond the COVID-19 pandemic. For this purpose, this paper examines the determinants of governments' fiscal support in response to the COVID-19 pandemic. The empirical analysis is based on the cross-sectional data estimations from 129 developed and developing countries. The estimation results indicate that a higher level of uncertainty related to COVID-19 (measured by the World Pandemic Uncertainty Indices) is positively related to fiscal support. Besides, countries with a higher total population and population over 65 years and older provide higher fiscal support. These results are valid when considering the developed countries separately. Policy implications for public finances during the COVID-19 pandemic are also discussed.

## Introduction

Decreasing the spread ratio of the COVID-19 virus is the priority of policymakers since COVID-19 has a higher death ratio than the common flu. It also creates massive pressure on the health system. Therefore, most countries have implemented various mitigation measures, such as the closure of public areas, restaurants, shopping centers, and schools. Some countries have introduced lockdown policies to decrease the spread ratio of COVID-19 (1, 2). However, these policy implications created tremendous uncertainty in economic policies (3). According to Baker et al. (4, 5), the COVID-induced economic uncertainty is one of the leading uncertainties that the modern economy has faced. This issue is related to the evidence that the COVID-19 crisis has caused both supply and demand-side shocks (6). The mitigation measures declined household consumption and production (7); thus, they created external shocks in demand and supply (8). These measures included business cash-flow and income-raising household measures (9, 10) and have distorted tax revenues (11).

At this stage, policymakers aim to enhance consumption and investment by providing stimulus packages. These stimulus packages include fiscal support measures, which have different public financial implications during the COVID-19 pandemic. Most countries have adopted a mixed approach of fiscal support tools, such as reducing taxes, direct benefits, loan guarantees, asset purchase or debt assumptions, equity injections, and quasi-fiscal operations (12).

There are previous papers that examine the determinants of fiscal policies during the COVID-19 pandemic. For instance, Benmelech and Tzur-Ilan (12) examine fiscal policy determinants during the COVID-19 pandemic. According to their findings, credit rating is the most important driver of fiscal support during the COVID-19 pandemic. High-income economies, which have a lower level of interest rates, provide non-conventional monetary policy implications. As a result, the lack of a solid credit rating and higher interest rates have been tagged as lower fiscal support during the COVID-19 pandemic. Finally, the authors observe that high-income economies provide a greater fiscal support level than middle-income and low-income economies. Following this evidence, we separately examine the case of developed countries, and the selection is based on the definition of high-income economies. Faria-e-Castro (13) theoretically examines the different types of fiscal policies on economic activity in the United States. The author finds that unemployment insurance benefits and liquidity assistance programs effectively stabilize households' income. Bredemeier et al. (14) also theoretically show that fiscal policies, particularly the decline in labor taxes, could increase total employment in different sectors during the COVID-19 crisis.

Furthermore, Kaplan et al. (15) show that economic uncertainty in pandemic-related developments is significantly correlated with financial vulnerability. Besides, fiscal policy responses to the COVID-19 pandemic create an uneven distribution of economic welfare costs. Siddik (16) investigates the determinants of stimulus packages in 168 countries and finds that the median age and health indicators (number of hospitals, beds per capita, and health care expenditures) are the main drivers of stimulus packages.

Given this backdrop, in this paper, we examine the determinants of governments' fiscal support in response to the COVID-19 pandemic. We utilize empirical analysis based on the cross-sectional data estimations from 129 developed and developing countries. We find that a higher level of uncertainty related to COVID-19 (measured by the World Pandemic Uncertainty Indices) is positively related to fiscal support. Besides, we observe that countries with a higher total population and population over 65 years and older provide higher fiscal support. These findings are robust when considering the developed countries separately.

The rest of the paper is organized as follows. The *Model, Data, and Estimation Procedures* section explains the details of the model, the data, and the estimation procedures. The *Empirical Results* section discusses the empirical results. The *Concluding Remarks* section provides the concluding remarks.

## Model, Data, and Estimation Procedures

In this paper, we estimate the following model via a cross-sectional data method in 129 developing and developed countries:

(1) $$\begin{array}{r} {FISCAL_{i}=\beta_{0}+\beta_{1}X}_{i}+\varepsilon_{i} \end{array}$$

where *FISCAL*_*i*_ is the fiscal support, *X*_*i*_ is the set of explanatory variables (per capita GDP, population, human development index, life expectancy, age 65 and older, and the World Pandemic Uncertainty Indices) in country *i*, and ε_*i*_ is the error term in the estimation.

Fiscal support is the total fiscal support, and it is the sum of the following supports: spending or foregone revenues (health sector and non-health sector) and liquidity support (asset purchase or debt assumptions, equity injections, guarantees, loans, and quasi-fiscal operations). Total fiscal support is defined as the share of gross domestic product (GDP). These data were obtained from the International Monetary Fund (IMF) (17) website (https://www.imf.org/en/Topics/imf-and-covid19/Fiscal-Policies-Database-inResponse-toCOVID-19).

We use several explanatory variables. For instance, we use the per capita GDP [measured by the Purchasing Power Parity (PPP) $ prices], population (in millions), the human development index (index from 0 to 1), life expectancy (in years), and age 65 and older (% of total population). These data were obtained from Hasell et al. (18) at the website (https://github.com/owid/covid-19-data/tree/master/scripts/scripts/testing).

Furthermore, we use the World Pandemic Uncertainty Indices (WPUI), which were provided by Ahir et al. (19) at the website (https://worlduncertaintyindex.com/data/). These indices provide the number of articles about the COVID-19 pandemic across the countries. The WPUI is provided by searching for words related to uncertainty in the COVID-19 pandemic country reports in the Economist Intelligence Unit (EIU) dataset. At this stage, a greater value of the WPUI provides a higher uncertainty related to the COVID-19 pandemic. The WPUI is used in the logarithmic form.

The fiscal support data are based on the total fiscal support from January 2020 to October 2020. Besides, the WPUI is the average of the values in 2020Q1, 2020Q2, and 2020Q3. Other variables are provided by Hasell et al. (18) at the cross-country level. The estimation procedures are utilized in 129 countries via cross-sectional data estimation. The selection of the countries is related to the availability of the data. Besides, we follow World Bank (20) and consider 39 developed countries^1^ whose per capita income is higher than $12,536 and above. Therefore, there are other 90 low-income and middle-income economies^2^ in the dataset, whose per capita income is lower than the related threshold. The descriptive statistics are reported in Table 1 for all countries in the dataset.

**Table 1:** Descriptive statistics: all countries.

**Variable**	**Definition**	**Tag**	**Mean**	**Standard deviation**	**Minimum**	**Maximum**	**Observation**
Fiscal support (total)	% of total GDP	FISCAL	6.676	7.268	0.100	39.20	129
Per capita GDP (PPP $)	Log form	Log GDPC	9.203	1.247	6.494	11.66	129
Population (millions)	Log form	Log POP	16.68	1.368	14.45	21.08	129
Human development index	Index from 0 to 1	HDI	0.709	0.161	0.354	0.953	129
Life expectancy	years	LIFE_EXP	72.56	7.941	53.28	84.63	129
Age 65 and older (ratio)	% of total population	AGED_65 OLDER	9.059	6.570	1.144	27.05	129
World Pandemic Uncertainty Indices	Index	Log WPUI	5.714	0.168	5.236	6.242	129

Finally, we provide a correlation matrix in Table 2 for all countries in the dataset.

**Table 2:** Correlation matrix: all countries.

**Indicator**	**FISCAL**	**Log GDPC**	**Log POP**	**HDI**	**LIFE_EXP**	**AGED_65 OLDER**	**Log WPUI**
FISCAL	1.000	–	–	–	–	–	–
Log GDPC	0.516	1.000	–	–	–	–	–
Log POP	0.140	−0.052	1.000	–	–	–	–
HDI	0.563	0.857	−0.067	1.000	–	–	–
LIFE_EXP	0.523	0.856	−0.018	0.824	1.000	–	–
AGED_65 OLDER	0.676	0.685	−0.082	0.784	0.749	1.000	–
Log WPUI	0.109	−0.475	−0.247	−0.488	−0.413	−0.393	1.000

The findings in Table 2 show the positive correlation between fiscal support and all right-side variables. Furthermore, the WPUI is negatively correlated with the remaining explanatory variables. There is a negative correlation between per capita GDP and population. In contrast, per capita GDP correlates with the human development index, life expectancy, and population of 65 years and older. However, the population is negatively correlated with the human development index, life expectancy, and 65 years and older population. Finally, there are positive correlations among the human development index, life expectancy, and 65 years and older population.

## Empirical Results

In Table 3, the results of the cross-sectional data estimations for 129 countries are reported.

**Table 3:** Cross-sectional data estimations for all countries: fiscal support in response to the COVID-19 pandemic.

**Indicators**	**(1)**	**(2)**	**(3)**	**(4)**	**(5)**	**(6)**
Log GDPC	3.009^***^ (0.442)	3.060^***^ (0.436)	1.879 (1.469)	2.123 (1.536)	1.001 (1.454)	1.077 (1.421)
Log POP	–	0.889^**^ (0.397)	0.971^**^ (0.381)	1.001^**^ (0.386)	1.067^***^ (0.341)	0.857^**^ (0.343)
HDI	–	–	39.87^***^ (11.37)	46.33^***^ (16.15)	0.118 (16.23)	4.933 (15.97)
LIFE_EXP	–	–	–	−0.101 (0.180)	−0.107 (0.159)	−0.141 (0.156)
AGED_65 OLDER	–	–	–	–	0.731^***^ (0.122)	0.738^***^ (0.119)
Log WPUI	–	–	–	–	–	8.278^***^ (3.178)
Constant Term	−21.01^***^ (4.110)	−36.33^***^ (7.955)	−20.55^**^ (8.852)	−15.97 (12.02)	−19.24^*^ (10.63)	−64.82^***^ (20.35)
Countries	129	129	129	129	129	129
R-squared (Adjusted)	0.261	0.283	0.342	0.339	0.484	0.507

*The dependent variable is total fiscal support in response to the COVID-19 pandemic, % of GDP (FISCAL)*.

*Standard errors are reported in parentheses*.

*** *p < 0.01*,

** *p < 0.05*,

and

* *p < 0.10*.

The first column includes only a log of the per capita GDP. In the second column, a log of total population is also added. The third column considers the log of per capita GDP, the log of the total population, and the human development index. The fourth column also includes the variables in the third column as well as life expectancy. The fifth column considers the variables in the fourth column and the share of the 65 years and older population in the total population. Finally, the sixth column considers all variables in the fifth column and the log of the World Pandemic Uncertainty Indices.

The results indicate that the coefficients of the log of the total population, the share of the population of 65 years and older in the total population, and the World Pandemic Uncertainty Indices are statistically significant (at a 5% level at least) in each case. All of these variables are also positively related to fiscal support.

Next, we provide the results in 39 developed countries. According to the IMF (17) data, fiscal support in developed countries is significantly higher than the other remaining 90 countries. In Table 4, the findings of the cross-sectional data estimations in 39 developed countries are provided.

**Table 4:** Cross-sectional data estimations for developed countries: fiscal support in response to the COVID-19 pandemic.

**Indicators**	**(1)**	**(2)**	**(3)**	**(4)**	**(5)**	**(6)**
Log GDPC	0.323 (3.906)	0.362 (3.293)	6.010^*^ (3.371)	5.260 (3.374)	4.458 (4.657)	2.974 (4.645)
Log POP	–	4.213^***^ (1.051)	3.197^***^ (0.978)	3.056^***^ (0.971)	3.018^***^ (0.887)	2.633^***^ (0.901)
HDI	–	–	108.1^***^ (32.49)	64.65 (45.10)	0.972 (47.12)	26.03 (48.66)
LIFE_EXP	–	–	–	0.856 (0.625)	0.539 (0.582)	0.204 (0.606)
AGED_65 OLDER	–	–	–	–	0.816^***^ (0.293)	0.781^***^ (0.287)
Log WPUI	–	–	–	–	–	10.38^**^ (4.510)
Constant Term	16.70 (41.37)	−51.98 (38.85)	−71.57^**^ (34.85)	−107.6^**^ (43.35)	−141.1^***^ (41.38)	−172.2^***^ (44.90)
Countries	39	39	39	39	39	39
R-squared (Adjusted)	0.002	0.270	0.429	0.443	0.535	0.556

*The dependent variable is total fiscal support in response to the COVID-19 pandemic, % of GDP (FISCAL)*.

*Standard errors are reported in parentheses*.

*** *p < 0.01*,

** *p < 0.05*,

and

* *p < 0.10*.

Like the structure in Table 3, the first column considers only the per capita GDP log. In the second column, the log of the total population is also included. The third column uses a log of per capita GDP, a log of the total population, and the human development index. Besides, the fourth column considers all variables in the third column as well as life expectancy. The fifth column includes the fourth column variables and the share of the 65 years and older population in the total population. Finally, the sixth column considers all variables in the fifth column and the log of the World Pandemic Uncertainty Indices.

The results show that the coefficients of the log of the total population, the share of the population of 65 years and older in the total population, and the World Pandemic Uncertainty Indices are statistically significant (at a 5% level at least) in each case. These indicators are also positively associated with the fiscal support in developed countries. Overall, the fiscal support's baseline determinants are robust when considering the countries at different income levels.

## Concluding Remarks

Fiscal support measures have different implications for public finances in the near term and beyond the COVID-19 pandemic. This paper investigated potential determinants of governments' fiscal support in response to the COVID-19 pandemic. We used cross-sectional data in 129 developed and developing countries. We found that a greater level of uncertainty related to COVID-19 measured by the World Pandemic Uncertainty Indices is positively associated with fiscal support. Moreover, the countries with a higher total population and the population over 65 years and older provide higher fiscal support to the COVID-19 pandemic. These findings remain unchanged when we focused on the developed countries.

Our results indicate that fiscal support is higher in countries that have higher uncertainty related to COVID-19. This evidence indicates that World Pandemic Uncertainty Indices are a suitable measure to capture the economic effects of COVID-19. The total population is positively related to fiscal support. Given that the spread ratio of the new type of coronavirus is higher in crowded countries than in small countries, this evidence contradicts theoretical expectations. Similarly, countries with an elderly population (measured by the population over 65 years and older) provide more fiscal support related to COVID-19.

Interestingly, our results indicate that the fiscal support is not significantly related to the per capita income or development indicators. This evidence shows us that governments have looked at the COVID-19-related uncertainty outcomes and want to protect the whole population in general, older people in particular. Other specific groups (e.g., job losers) can be included in the fiscal stimulus packages.

Note that our findings are limited to total stimulus packages. Future papers can focus on the fiscal support determinants in different sectors (e.g., education and health) in developed and developing economies. Another research plan can examine fiscal support determinants in commodity exporter and commodity importer countries since some countries' fiscal situation has been worsened by commodity price shocks.

## Data Availability Statement

The original contributions presented in the study are included in the article/supplementary material, further inquiries can be directed to the corresponding author/s.

## Author Contributions

S-KL conceputalization and econometric analysis. XL conceputalization and writing. XL review and writing. All authors contributed to the article and approved the submitted version.

## Conflict of Interest

The authors declare that the research was conducted in the absence of any commercial or financial relationships that could be construed as a potential conflict of interest.

## Footnotes

^1^Australia, Austria, Belgium, Canada, Chile, Croatia, the Czech Republic, Denmark, Finland, France, Germany, Greece, Hungary, Ireland, Israel, Italy, Japan, Korea Republic, Kuwait, Latvia, Lithuania, the Netherlands, New Zealand, Norway, Poland, Portugal, Qatar, Romania, Saudi Arabia, Singapore, Slovakia, Slovenia, Spain, Sweden, Switzerland, the United Arab Emirates, the United Kingdom, the United States, and Uruguay.

^2^Afghanistan, Albania, Algeria, Angola, Argentina, Armenia, Azerbaijan, Bangladesh, Benin, Bolivia, Bosnia and Herzegovina, Botswana, Brazil, Bulgaria, Burkina Faso, Burundi, Cambodia, Cameroon, Central African Republic, Chad, China PR, Colombia, Congo Republic, Costa Rica, Côte d'Ivoire, Democratic Republic of the Congo, Dominican Republic, Egypt, El Salvador, Ethiopia, Gabon, Georgia, Ghana, Guatemala, Guinea, Guinea-Bissau, Haiti, Honduras, India, Indonesia, Iraq, Iran, Jamaica, Jordan, Kazakhstan, Kenya, Kyrgyz Republic, Lesotho, Liberia, Libya, Madagascar, Malawi, Malaysia, Mali, Mauritania, Mexico, Moldova, Mongolia, Morocco, Mozambique, Myanmar, Namibia, Nepal, Nicaragua, Niger, Nigeria, Pakistan, Panama, North Macedonia, Paraguay, Peru, Philippines, Russia, Rwanda, Senegal, Sierra Leone, South Africa, Sri Lanka, Sudan, Tajikistan, Thailand, Togo, Tunisia, Turkey, Uganda, Ukraine, Uzbekistan, Vietnam, Zambia, and Zimbabwe.
